# A Label Free Disposable Device for Rapid Isolation of Rare Tumor Cells from Blood by Ultrasounds

**DOI:** 10.3390/mi9030129

**Published:** 2018-03-15

**Authors:** Itziar González, Julie Earl, Luis J. Fernández, Bruno Sainz, Alberto Pinto, Rosa Monge, Sonia Alcalá, Adela Castillejo, Jose L. Soto, Alfredo Carrato

**Affiliations:** 1 Institute of Physical Technologies, Consejo Superior de Investigaciones Científicas, Serrano 144, 28006 Madrid, Spain; alberto.delcorral@csic.es; 2 Hospital Universitario Ramón y Cajal, CIBERONC & IRYCIS, 28034 Madrid, Spain; julie.earl@live.co.uk (J.E.); acarrato@telefonica.net (A.C.); 3 Universidad Zaragoza UNIZAR, Pedro Cerbuna 12, 50009 Zaragoza, Spain; luisf@unizar.es (L.J.F.); rosmonge@unizar.es (R.M.); 4 Department of Biochemistry, Universidad Autónoma de Madrid (UAM) Instituto de Investigaciones Biomédicas “Alberto Sols” CSIC-UAM, Arzobispo Morcillo 4, 28029 Madrid, Spain; bruno.sainz@uam.es (B.S.); sonia.alcala@uam.es (S.A.); 5 Laboratorio Genética Molecular. Hospital Gral Univ. Elche, Carrer Almazara, 11, 03203 Elche, Spain; castillejo.ade@gmail.com (A.C.); soto_jos@gva.es (J.L.S.)

**Keywords:** tumor cell isolation, ultrasounds, low-cost, microfluidics, plate acoustic waves

## Abstract

The use of blood samples as liquid biopsy is a label-free method for cancer diagnosis that offers benefits over traditional invasive biopsy techniques. Cell sorting by acoustic waves offers a means to separate rare cells from blood samples based on their physical properties in a label-free, contactless and biocompatible manner. Herein, we describe a flow-through separation approach that provides an efficient separation of tumor cells (TCs) from white blood cells (WBCs) in a microfluidic device, “THINUS-Chip” (Thin-Ultrasonic-Separator-Chip), actuated by ultrasounds. We introduce for the first time the concept of plate acoustic waves (PAW) applied to acoustophoresis as a new strategy. It lies in the geometrical chip design: different to other microseparators based on either bulk acoustic waves (BAW) or surface waves (SAW, SSAW and tSAW), it allows the use of polymeric materials without restrictions in the frequency of work. We demonstrate its ability to perform high-throughput isolation of TCs from WBCs, allowing a recovery rate of 84% ± 8% of TCs with a purity higher than 80% and combined viability of 85% at a flow rate of 80 μL/min (4.8 mL/h). The THINUS-Chip performs cell fractionation with low-cost manufacturing processes, opening the door to possible easy printing fabrication.

## 1. Introduction

The presence of circulating tumor cells (CTCs) in peripheral blood has been associated with a reduced progression survival in many cancer types [1,2]. These cells have become established as biomarkers of prognosis and may be useful as an early indicator of tumor spread, as invasive but localized tumors may shed CTCs into the bloodstream before the establishment of a metastasis [3,4,5]. Liquid biopsies offer an alternative to taking tumor samples as they provide information of the disease from a blood sample replacing the invasive techniques. Detecting, isolating, and analyzing CTCs has the potential to improve diagnosis, allow prognostic monitoring, and enable targeted treatment strategies that are based on the metastatic cells mainly responsible for cancer mortality. However, they are challenging to isolate [6,7]. Several attempts have been developed to address this, but the performance of new devices for high-throughput CTC detection and isolation is currently insufficient. These systems must meet five objectives concerning the highest efficiency, purity, sensitivity, cell viability and throughput-speed to exploit the full potential to isolate viable CTC of a high-purity for clinical applications. New low-cost approaches are required for translational clinic applications of diagnostic and monitoring. 

Cell sorting on microfluidic devices provides numerous advantages over conventional methods by reducing the size of necessary equipment and simplifying the complex protocols commonly associated with cell sorting. Different mechanisms are selected as the base working principle of the lab-on-chip platforms. Many of the current strategies for detecting CTC in peripheral blood use differential physical cell properties to distinguish CTC from blood cells, including their size (epithelial cells), shape, deformability and differences in density. These properties are separated by size [8,9,10,11,12,13,14,15], using spiral channelization [16,17,18,19], channels that incorporate contraction/expansion reservoirs for pinch alignment of the cells, micropillars [20,21,22,23], micro-scale vortices [24,25], micron-sized gaps [26], serpentine microfluidic channels [27,28] or membrane microfilters+ [29] with pore diameters chosen to have the dimension between the diameters of cancer cells and blood cells. Other specific properties of cells are also considered to perform their sorting, such as their electric charge or migratory properties. 

The acoustic sorting method offers a means to separate cells based on their physical properties in a label-free, contactless, and biocompatible manner. A successful separation of cultured cancer cells from WBCs with acoustic-based methods was recently demonstrated in some microfluidic platforms using Bulk acoustic waves (BAW) [30,31] and Surface acoustic waves (SAW) [32,33] applied either on cultured cancer cells or tumor cells in spiked blood samples respectively. These methods base their actuation on processes of differentiated cell enrichment induced by ultrasounds on flowing samples, involving mass transfer processes between parallel flows in some cases. The acoustic force is used to drive and collect target cells operating on size, density and compressibility of different cell populations [34,35]. The acoustic technology for sorting purposes works at power intensities and frequencies similar to the ultrasonic imaging, with a little impact on the viability (high biocompatibility) [36]. It presents clinical advantages such as the fact that the media in which cells are cultured and separated does not need to be modified, thus no labeling is required. This ultimately maximizes the potential of CTCs to be maintained in their native states, cultured, and analyzed in vitro or ex vivo. This non-contact and label-free separation of tumor cells from blood enables their recovery regardless of their molecular profile. It offers the potential of early detection of cancer and micro-metastasis as well as noninvasive monitoring of the cancer patients undergoing treatment. 

BAW approaches for cell sorting purpose base their actuation either on 1D/2D resonances established inside the channel of treatment between the sidewalls [31] or 3D resonances across the whole chip structure [30]. The former requires rigid materials (usually glass or silicone) and strict parallelism of the channel walls for the establishment of the standing wave. On the contrary, recently developed 3D chip resonators allow the use of structural materials of a low acoustic impedance and stiffness, such as polymers. These structures do not impose strict restrictions in the channel width, as reported previously by our group [30,37]. These devices are suitable for bio-applications due to their high bio-compatibility and low-cost manufacture. In addition, recent development of specific microfabrication methods based on 3D printers open the door for mass production of these microfluidic devices of simple design. 

The SAW-based separators also allow polymeric materials and a highly specific spatial manipulation of single cells without restricted specifications for the channel width. However, their operating frequencies are limited to approximately one order of magnitude higher than BAW devices due to the physical limitations of their interdigital transducers (IDT), which are defined by the frequency of work and thickness restrictions for the establishment of the surface wave (a much smaller wavelength than the chip thickness is mandatory in SAW devices). Published data on SAW separators refer to working frequencies over 9 MHz [32,38]. 

In this paper, we introduce for the first time the concept of plate-vibrations in the development of a new polymeric acoustophoretic chip actuated by a single piezoelectric transducer to perform high-throughput isolation of tumor cells from flowing blood samples: “THINUS-Chip” (Thin-Ultrasonic-Separator-Chip). It offers the advantages of BAW or SAW microseparators together and overcomes their respective disadvantages: it allows the use of polymeric materials for the chip structure, with lower manufacturing cost than the BAW devices and lower frequencies than those required by the SAW isolators, i.e., close to 1 MHz. 

A prerequisite of the BAW separators is that the channel dimensions are in concert with the ultrasound frequency [34]. Resonance occurs when one dimension of the channel can support an integer number of half-wavelengths in the suspending fluid. Unlike these, our THINUS-Chip separator allows the use of SU-8 polymer for the chip structure as it is based on a Plate Acoustic Wave-actuation (PAW) without rigidity requirements, and allows frequencies as low as those of BAW devices. It is highly-reliable and performs the cell separation with a high-efficiency and fast fluidic actuation (allowing high flow rates of operation), as demonstrated in this article. In addition, its simple geometrical design and structural properties open a line for a near future easy printing. 

## 2. Materials and Methods

The THINUS-chip based cell separation relies on the establishment of typical modes of vibration of a plate that, at a specific frequency close to 1 MHz (*f* = 952 KHz), generate a pressure node within the channel at a desired location where the target cells collect before their isolation. The geometrical configuration of the chip associated to a very large surface/volume ratio together with a thickness slightly smaller than a quarter of the wavelength at his frequency provides the Plate-like actuation of the plastic structure of the chip. This configuration fulfills the theoretical conditions of the models developed first by Mindlin and later by other authors to study free and forced vibrations of thick and moderately thick rectangular plates with different boundary conditions and thicknesses [38,39]. At the frequency selected of 952 KHz, the chip vibration generates a pressure patterning inside the channel with a planar pressure node in its center, parallel to the channel walls.

### 2.1. Working Principle for High-Throughput Separation

Each cell circulating inside can be assumed as spherical compressible particle, with a volume “*V_c_*” much smaller than the acoustic wavelength “*λ*”, experiences a primary acoustic radiation generated by the acoustic standing wave with amplitude *P*_0_ according to its specific properties [40]:

(1) $$FR_{c}=\frac{\pi P_{0}^{2}V_{c}\beta_{l}}{2\lambda }\varphi \left(\rho_{c},\beta_{c},\rho_{l},\beta_{l}\right)sin\;\left(\frac{4\pi x}{\lambda }\right)$$

with the acoustic contrast factor $\varphi \left(\rho_{c},\beta_{c},\rho_{l},\beta_{l}\right)=\frac{5\rho_{c}-2\rho_{l}}{2\rho_{c}+\rho_{l}}-\frac{\beta_{c}}{\beta_{l}}$. It defines the relationship between the densities and adiabatic compressibilities of cells (*ρ_c_* and *β_c_*) and liquid (*ρ_l_* and *β_l_*), respectively. The distance from the cell to the node of pressure established inside the channel is defined by “*x*”. The sign of *ϕ* indicates the motion of the cells, either toward the nodes (*ϕ* > 0) or to the antinodes in the standing wave (*ϕ* < 0), respectively. The acoustic contrast factor is positive for the various blood cells and cancer cells suspended in isotonic aqueous solution such as PBS (phosphate buffered saline) or standard cell culture media, so they are expected to collect at the pressure nodes. 

The velocity of the cells moving towards a pressure node can be obtained by equating the average ultrasonic force acting to move cells into hands with Stoke’s drag force from the quiescent liquid, $F_{Dx}=-6\pi \eta R_{p}u_{pz}$, neglecting inertial effects for micrometer-sized spherical particles:

(2) $$u_{x}=\frac{P_{0}^{2}V_{p}\beta_{l}}{12\lambda \eta R_{p}}\varphi \left(\rho_{p},\rho_{l},\rho \beta_{p},\beta_{l}\right)sin\left(2kx\right)$$

The particle position and its trajectory at any time “*t*” can be derived from this expression as:

(3) $$x\left(t\right)=\frac{1}{k}arctan\left\{tan\left(kx\left(0\right)\right)e^{\frac{4\varphi }{3}{\left(kR_{p}\right)}^{2}\frac{E_{ac}}{\eta }t}\right\}$$

with the acoustic energy density: $E_{ac}=\;\frac{p_{0}^{2}}{4\rho_{0}\cdot c_{0}^{2}}$. The time required by the cells to reach a certain position “*x*” driven by the acoustic radiation force from any initial position *x*(*t* = 0) in the standing wave can also be derived from Equations (1) and (2) as:

(4) $$t=\frac{3\eta }{4\varphi \left(kR_{c}^{2}\right)E_{ac}}ln\left[\frac{tan\left[k\cdot x\left(t\right)\right]}{tan\left[k\cdot x\left(0\right)\right]}\right]$$

Cells with different physical properties (size, density and compressibility) experience different amplitudes of the acoustic radiation force. It allows discriminated effects associated to slightly different sized cells, even of few microns. The ratio of volumes of two cells differing in their radii $R_{1}$ and $R_{2}$ differing in size Δ*R*, $(R_{2}=R_{1}+\Delta$*R)* is:

(5) $$\frac{V_{c2}}{V_{c1}}=\frac{{\left(R_{1}+\Delta R\right)}^{3}}{R_{1}^{3}}\;\sim 1+3\frac{\Delta R}{R_{1}}+3{\left(\frac{\Delta R}{R_{1}}\right)}^{2}+{\left(\frac{\Delta R}{R_{1}}\right)}^{3}$$

**Figure 1:**
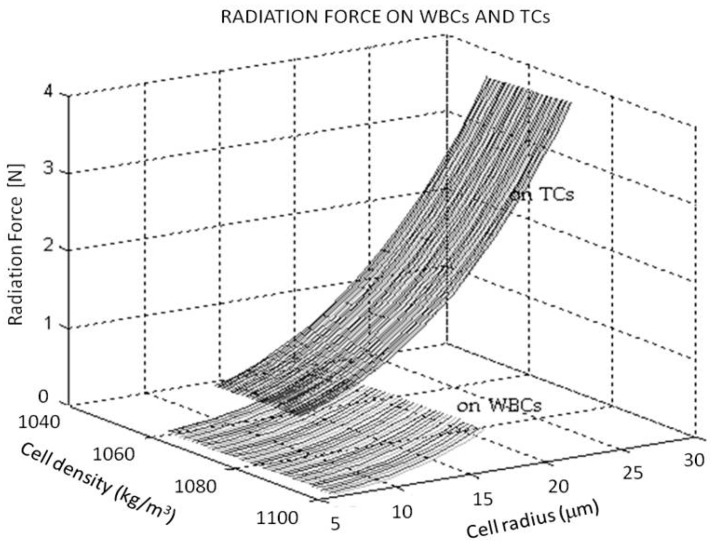
Estimate of the radiation force exerted on WBCs and TCs, respectively, at the same acoustic conditions.

It is reduced to a linear relationship for small size difference of $\Delta R\leq \frac{R_{1}}{10}$ (becoming negligible the nonlinear terms. In particular, it becomes $\frac{FR_{2}}{FR_{1}}\sim 1.3$ when $\Delta =\frac{R_{1}}{10}$. Thus, a difference of size of few microns between two cells with similar density and compressibility generates different radiation forces on them. This parameter can be used as a tool for sorting or separation in bi-disperse or multi-disperse suspensions (containing two or more particle or cell populations). The size of epithelial circulating tumor cells TC and WBCs may differ by few microns (frequently over 5 μm). According to Equation (1), size, density and compressibility are three parameters that must be considered to compute the radiation force established on both types of cells. Figure 1 shows a numerical estimation of the radiation force expected on a TC and a WBC, respectively, exposed to ultrasounds obtained running Matlab (MathWorks, Natick, MA, USA) with the same acoustic conditions and assuming the ranges of variability for the density and size of both type of cells given in Table 1.

These numerical results show the TC more susceptible to the acoustic field over a range of sizes, regardless of their density and compressibility within the range considered. They experience a stronger radiation force than WBCs exposed to the same acoustic wave pressure, despite of the higher density and lower compressibility of the WBCs. These results show the cell size as the most influential parameter in Equation (1) and explain the ability of the ultrasounds to effectively isolate TC from blood samples. 

**Figure 2:**
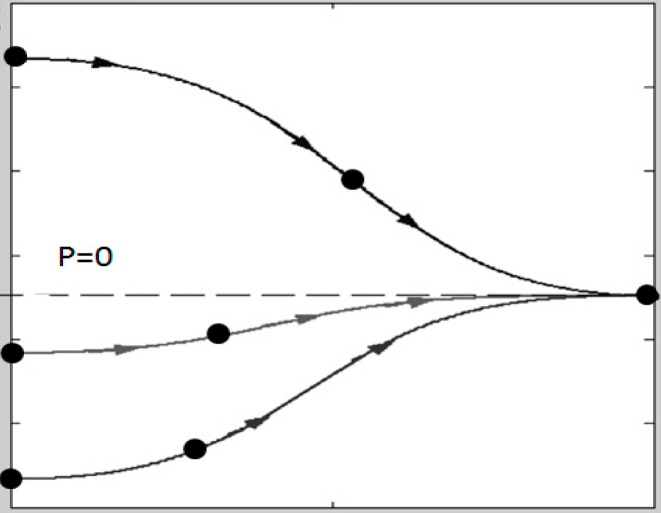
Numerical estimate (Matlab) of trajectories of three equal particles approaching a pressure node from different initial positions while circulating in a transverse direction.

To calculate the motion of a cell of mass *m_p_*, the force terms are summed to give accelerations along the two dimensions of the channel cross section, defined on the *x*- and *z*-axes:

(6) $$\frac{du_{px}}{dt}=\frac{\sum F_{x}}{m_{p}}=\frac{F_{Dx}+F_{radx}}{m_{p}}$$

(7) $$\frac{du_{pz}}{dt}=\frac{\sum F_{z}}{m_{p}}=\frac{F_{Dz}+F_{radz}+F_{Bz}}{m_{p}}$$

where *u_px_* and *u_pz_* refer to the particle velocity components along the two axis of the channel cross section, *F_radx_* and *F_radz_* the radiation force components acoustically induced on the *x*- and *y*-dimensions, *F_Dx_* and *F_Dz_* the fluid drag forces acting on the particles, and *F_B_* the buoyancy force exerted opposite to the gravitational force. *F_Dx_* and *F_Dz_* are defined along the *x*- and *z*-directions, respectively:

(8) $$F_{Dx}=6\pi \eta R_{p}\left(u_{px}-u_{lx}\right)$$

(9) $$F_{Dz}=6\pi \eta R_{p}\left(u_{pz}-u_{lz}\right)$$

for low Reynolds numbers Re < 0.2, where η is the dynamic viscosity coefficient, *u_lx_* and *u_ly_* are the *x-* and *y*-components of the laminar flow velocity of the fluid associated to the parabolic profile within a microfluidic channel:

(10) $$u_{lx}=\frac{6U}{w^{2}}\left(wx-x^{2}\right)$$

(11) $$u_{lz}=\frac{6U}{h^{2}}\left(hz-z^{2}\right)$$

and “*h*” and “w” are the channel height and width, respectively. A numerical resolution of Equations (6) and (7) provides particle trajectories from any distance to the nearest pressure node and the time required to reach the pressure node, depending on the different parameters involved the equations of particle motion. The 4th order Runge–Kutta method is a suitable tool for the numerical solver. Figure 2 shows a numerical estimate of the trajectories of three equal particles in a plane standing wave travelling toward the pressure node from three different initial positions while circulating in a transverse direction. 

A suitable combination of the channel length and flow velocity keeps the particles circulating in the channel for long enough to reach the pressure node from any distance. Higher flow rates elongate the particle trajectories, requiring longer channel lengths to reach the pressure node. This implies a compromise between hydrodynamic and acoustic conditions (flow rate/frequency and pressure amplitude) considering the particle properties of the particles together with those of the host fluid (size, density and compressibility ratios as well as the fluid viscosity).

### 2.2. Experimental

#### Acoustophoretic Device

**Figure 3:**
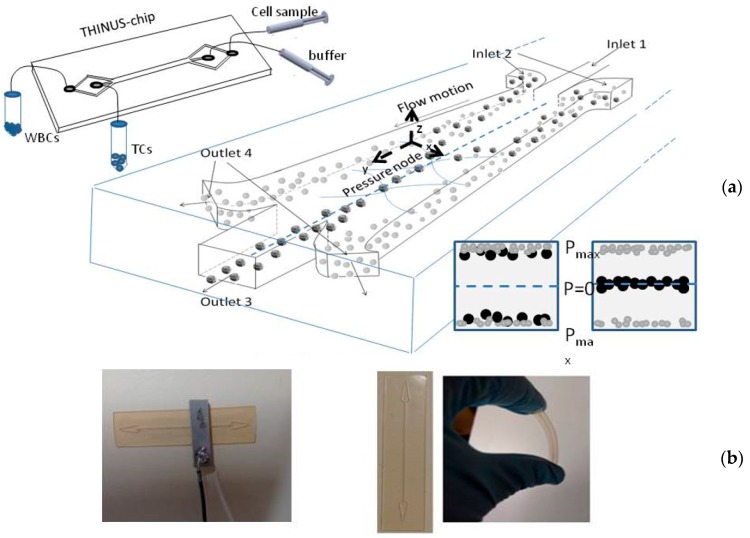
(**a**) A schematic view of the present cancer cell isolation THINUS-chip, with a collection of the bigger particles along the center of the channel, while the small particles continue their flow motion undisturbed by the acoustic field, and (**b**) the fabricated cancer cell isolation chip.

The THINUS-Chip was made up of a rectangular thin piece (10 mm × 40 mm × 250 µm) made up of epoxy SU-8 containing a straight channel with two inlets and two outlets symmetrically displayed (scheme and photo of the device are shown in Figure 3) and layered over another SU-8 substrate without channelization and identical dimensions. This polymer is highly hydrophobic and presents a good biocompatibility for the application. The channel had a rectangular cross section of 400 µm (width) × 150 µm (height) and a length of 25 mm.

The THINUS-chip was actuated by a small piezoelectric ceramic Ferroperm pz26 (Ferroperm, Krisgard, Denmark) of a rectangular area (8 × 5 × 1.5 mm^3^) with a thickness-mode resonance at 1 MHz. It was attached to the chip top covering an area partially occupied by the channel. The piezoelectric ceramic was activated by a function generator (Agilent 33220A, Agilent Technologies Inc., Santa Clara, CA, USA) equipped with a power amplifier (E&I RF linear broad Amplifier 240 L, Research Blvd. Rochester, NY, USA). The voltage supplied to the transducer was measured and controlled by an oscilloscope (Tektronix TDS 3034B, Tektronix UK Ltd., Bracknell, UK). 

The thickness of the chip was 500 µm, approximately a quarter of the acoustic wavelength in SU-8 at a frequency close to 1 MHz. It was reduced to 150 µm over the channel (~λ/12) and 250 µm below it (~λ/8), inhibiting any resonance in the z-direction within this area. The small thickness made the chip highly flexible, as shown in the photograph of the Figure. At a specific frequency *f* = 952 kHz, we found a mode of vibration of the chip generating a pressure node in the center of the channel, parallel to the sidewalls. The strategy of positioning the node along this axis (where the flow velocity achieved a maximum amplitude) optimized the combined pressure-driven flow and acoustic actuation, promoting specific lateral deviations in the cell trajectories during their flow motion along the channel for their transfer to the free-cells buffer. 

In the current configuration, the channel has been performed centered within the chip, symmetrically distanced to its lateral edges, which are subjected to identical boundary conditions of free external walls. Without a requirement of high reflectivity of the channel walls for the establishment of a resonance between them, the THINUS-chip allows the use of the polymers for the chip structure resonance (materials with low acoustic impedances). In particular, epoxy SU-8, with a low acoustic impedance *Z*_SU-8_ = 2.21 MRayls, which is relatively close to that of the fluid (*Z*_Blood_ = 1.66 MRayls), was used.

The samples were infused through the lateral Inlet 2 to flow beside the walls in parallel to a buffer free of cells infused through the central Inlet 1 and flowing along the central area of the channel. Under the action of the ultrasounds, certain target particles were driven toward this area where a pressure node was established and continued their circulation until the end of the channel to leave the device through the central Outlet 3. Meanwhile, the other particles, less susceptible to the acoustic wave, remained flowing without alterations and were extracted through the lateral Outlets 4.

**Figure 4:**
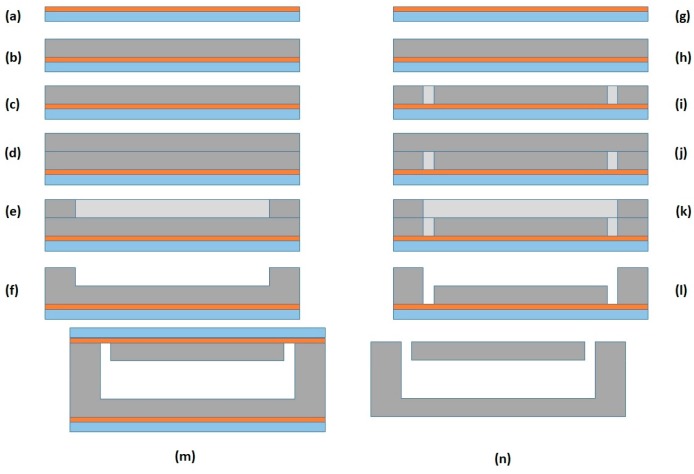
Fabrication process with: (**a**) pyrex-kapton bonding; (**b**) a 90 µm SU-8 layer; (**c**) ground definition; (**d**) 90 µm SU-8 layer; (**e**) microchannels definition; (**f**) wafer development; (**g**) pyrex-kapton bonding; (**h**) a 90 µm SU-8 layer; (**i**) inlets and outlets definition; (**j**) 90 µm SU-8 layer; (**k**) microchannels definition; (**l**) wafer development; (**m**) wafer bonding; and (**n**) final release of the devices.

**Microdevice fabrication.** For the fabrication of the microfluidic chip, SU-8 based technology was applied (Figure 4). The process starts with the temporary bonding of a thin kapton film (125 µm) on top of a pyrex substrate (a). Kapton was used because of its low adhesion to SU-8, allowing the releasing of the devices from the substrate when their fabrication is finished. Once the kapton film was fixed to the substrate, a 60 µm thick SU-8-50 layer was deposited on top of it. After every spinning step, a soft-bake treatment was performed. All soft-bake steps were performed by heating the wafer up to 65 °C for 30 min, followed by a cooling step down to room temperature. Then, another spinning of a 20 µm thick layer was performed followed by a new soft-bake step. As a result, a 90 µm thick layer was obtained (b). The increased thickness from the expected 80 µm was caused by the difference in surface friction: the first layer was spun on top of a kapton film, while the second was spun over SU-8 material, increasing its expected thickness. Next, a 140 mJ/cm^2^ exposure dose was used to pattern the floor layer of the device using a 365 wavelength lamp, followed by a post-bake step, heating of the wafer up to 6 °C for 15 min and cooling it down to room temperature (c). After this step, one layer of 60 µm and one of 20 µm were spun followed by a soft-bake process (d). An exposure of 140 mJ/cm^2^ was then applied using the mask which defines the microchannels (e). Then, a post-bake was performed followed by a development step to remove the unexposed SU-8 (f). The development consisted on an immersion of the exposed wafer into a SU-8 developer for 8 min, followed by a rinsed in isopropanol, DI H_2_O and a drying step using nitrogen. As a result, open microchannels with half of the total desired height were fabricated. To close the microchannels reaching the required height, another wafer was processed. First, a 90 µm thick SU-8 layer was processed (spun and soft-baked) on top of another kapton film temporary bonded to a pyrex wafer (g and h). Inlets and outlets were patterned by photolithography using the same exposure and baking parameters (i). Next, 90 µm thick layer was processed on top (j). This final layer was patterned using the mask which defines the microchannels, followed by a post-bake step (k). The wafer was finally developed (l). Then, both wafers (the bottom and the cover) were aligned and bonded to each other applying a pressure of 1 bar and a heating up to 90 °C (m). Finally, the bonded SU-8 devices were manually released from the kapton thanks to its low adhesion (n).

The sample-loading procedure of the THINUS-Chip was very simple. The samples were pumped into the chip via a high pressure syringe pump (Kd Scientific Syringe Pump, Boston/Cambridge, MA, USA), which allowed the fine control of insertion flow rates. One-milliliter samples were infused simultaneously with a particle-free buffer solution (distilled deionized water) through two 100 µm-width inlets, which converged in a common 400 µm-width channel of treatment to flow in parallel. The channel structures of very simple geometry avoided clogging problems from occurring when a blood sample passed through the microchannel and isolating single tumor cells (TCs). Once infused, the cells remained confined, flowing parallel in narrow paths close to the sidewalls around the buffer solution before the application of the acoustic field.

**Cell Sample Preparation**. Standard protocols and procedures were used to prepare human WBCs and Panc-1 cells for the experiments. Peripheral blood samples were obtained from healthy donors in the hospital Ramón y Cajal (Madrid, Spain). Mononuclear cells were separated from whole blood by gradient centrifugation using Ficoll. Red blood cells were lysed using Buffer EL (Erythrocyte Lysis Buffer, Qiagen) to reduce the blood viscosity, minimizing its behavior as non-Newtonian fluid. The human pancreas cancer cell line, Panc-1, was cultured in RPMI media supplemented with 10% serum under standard conditions. When at approximately 80% confluence, the cells were harvested using trypsin. Replicate samples of 300,000 Panc-1 cells spiked in 100 µL of PBS with 1 million mononuclear cells were used for the tumor cell isolation. Additional vials of 30,000 Panc-1 cells alone and 1 million mononuclear cells alone were prepared to calibrate the US chip. The Panc-1 tumor-cell blood/mononuclear cell (Ls) sample flowed in parallel with PBS (10% diluted in distilled water) along the channel during the acoustic treatment.

## 3. Results

### 3.1. Calibration of the Device with Bead Suspensions

To test the actuation of the THINUS-chip, dyed polystyrene beads (Dynoseeds Co. TS20 and CA6 Microbeads AS, Skedsmokorset, Norway) of 6 μm and 20 μm were used in a bi-disperse suspension. The solutions were prepared by diluting the particles in deionized distilled water using a concentration (*C*_v_ ~ 0.01%). These suspensions contained 50% of each size beads (*C*_v6_ ~ 0.005% and *C*_v20_ ~ 0.005%) for particle separation procedures. Total flow rates varying from 20 µL/min (1.2 mL/h) to 100 µL/min (6 mL/h) were tested in the experiments. 

**Figure 5:**
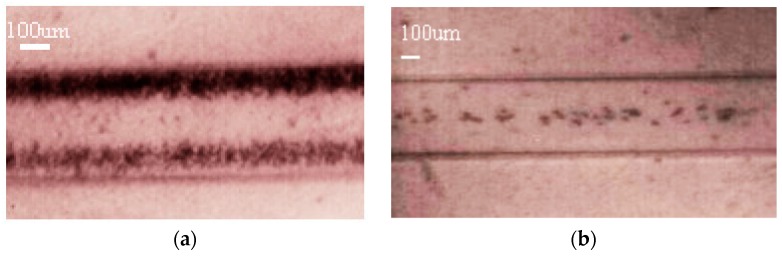
Filmed images of 6 µm- and 20 µm-sized polystyrene beads flowing in the channel at a flow rate *Q* = 100 µL/min: (**a**) without ultrasounds; and (**b**) exposed to the ultrasounds at *f* = 0.952 MHz. The bigger circulating particles (20 µm) collected along the central axis within the buffer while smaller particles kept flowing beside the sidewalls (images have poor quality due to the translucent polymer covering the top of the channel and the high speed of the particle flow).

Samples containing the beads were infused through the lateral Inlet 2 of the chip (Figure 3) to flow in parallel around the buffer flowing free of particles, which was injected through the Inlet 1. Once the ultrasounds were applied, different radiation forces were obtained on the two populations of particles, being dominant over the viscous drag force for the larger particles (20 µm-sized), which experienced lateral displacement towards the center of the channel. Conversely, the drag force dominated on the small particles (6 µm-sized) over the acoustic force, resulting in little lateral displacement; these small beads continued their flow motion near the sidewalls without apparent changes. Figure 5 shows two filmed images of these flowing suspensions before and during the actuation of the ultrasounds. The 6 µm- and 20 µm-sized beads circulate together beside the channel walls around a buffer free of particles before the ultrasonic treatment (Figure 5a). Once the ultrasounds were applied, the 20 µm-size beads were rapidly transferred to the center, where they continued to be collected along the channel (Figure 5b) and extracted through the central outlet, separated from the smaller 6-µm particles that left the channel through the lateral outlets. Thus, using the Plate Acoustic Wave principle of our elastic acoustophoretic separator, we were able to separate 6- and 20-μm polystyrene beads with a 96% ± 2% separation efficiency.

### 3.2. Actuation of the Ultrasounds on Tumor Cells and White Blood Cells

In the experiments, the TC showed a greater susceptibility to the acoustic field than the blood cells, agreeing with our theoretical expectations (see Figure 1). According to their physical properties, several cultured pancreas cancer line cells TCs (Φ_TC_ > 15 μm) were laterally entrained by the acoustic radiation force to reach the central node of pressure, keeping the smaller WBCs (Φ_WBC_ ≤ 15 μm) flowing beside the channel walls at flow rate of 20 µL/min ≤ *Q* ≤ 100 µL/min and certain voltages supplied to the expectated piezoelectric ceramic. These results also agree with other studies in the literature reporting acoustic separation of TCs from WBCs [35,36,37].

A threshold voltage of *V*_p-p_ (WBC) ~ 30 V required by the WBCs to collect along the center of the channel was found in the experiments for any of the flow rates tested below 120 uL/min. Over that voltage, they collected together with the epithelial tumor cells, making cell separation impossible. Thus, voltages lower than this threshold were supplied to the piezoelectric actuator to perform the TCs isolation, specifically a voltage of 28 V. Different flow rates were tested to find an optimal combination of hydrodynamic and acoustic conditions and thus achieve the highest efficiency of selective collection of TCs in the center for subsequent separate extraction of the WBCs.

The efficiency of isolation was defined as a quantified result of TC separation from WBCs. The enrichment percentage of tumor cells was calculated as the ratio of the numbers of the panc-1 tumor cells collected through the central outlet, divided by the same ratio at the inlet. 

TC and WBCs extracted through the different outlets were quantified by flow cytometry. The number of cells released from the channel through the central outlet and delivered to an Eppendorf^®^ collector (Eppendorf, Hamburg, Germany) was quantified in a Z2 Beckman Coulter Counter and classified by their size, and they compared to the total number of cells of identical size range extracted through all the outlets (this one and the lateral outlet) to determine the efficiency of extraction according to protocols followed by other authors:

(12) $$\eta_{CTs}\left(\%\right)=\frac{Number_{CTs}\left(central\;outlet\right)}{Number_{CTs}\;\left(central\;outlet+lateral\;outlets\right)}\;\times \;100$$

The degree of purity in the isolation of the CTs was determined inversely to percentage of white blood cells extracted through the central outlet with respect to the whole lateral and central outlets of the. It is a percentage complementary to the efficiency of extraction of the WBCs through the lateral outlets:

(13) $$\eta_{WBCs}\left(\%\right)=\frac{Number_{WBCs}\left(lateral\;outlets\right)}{Number_{WBCs}\;\left(central\;outlet+lateral\;outlets\right)}\;\times \;100$$

**Figure 6:**
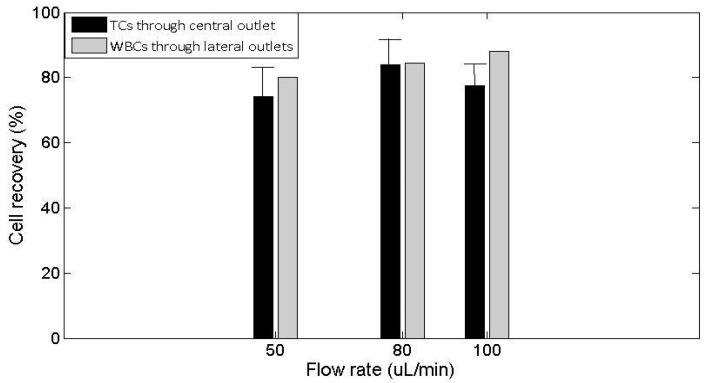
Results of the quantified capture efficiency of the cancer cell separation (%) of pancreas cancer cells (Panc-1) spiked in RBC-lysed blood samples obtained in the THINUS-Chip at *f* = 952 kHz and *V*_p-p_ = 28 V. The values given are means, the error bars denoting min and max values.

High efficiency levels of TCs separated from the WBCs by ultrasounds were achieved in the 20 experimental tests of our experiments: over 70% within the range of flow rates tested between 50 µL/min (3 mL/h) and 100 µL/min (6 mL/h), as shown in Figure 6 (see Figure S1). A percentage of 74 ± 1% of TC recovery through the central outlet was achieved at a flow rate of *Q* = 50 µL/min (3 mL/h), with a degree of purity (isolation) of 80.1%. An increase of the flow rate up to *Q* = 80 µL/min (4.8 mL/h) allowed a higher efficiency of the TC enrichment and extraction through the central outlet, up to 84.0 ± 8%, with a high purity degree of approximately 84.4%. A singular recovery of the TCs through the central outlet of 96% with a purity degree of isolation of 90% was achieved in one test with these flow rate conditions. At a higher flow rate, *Q* = 100 µL/min (6.0 mL/h), the efficiency on TC extraction reached 77% ± 6% through the central outlet with a degree of purity of 88%. The ability of the chip to perform TCs isolation decreased dramatically at higher flow rates and poorer results were obtained over this flow conditions. In particular, TC extraction below 68% and low purity rates (64%) were obtained at a flow rate of 200 µL/min. This was discarded for the analysis. 

Diverse cell aggregates were also present in our experiments, which experienced stronger radiation forces than the single cells proportional to their larger volumes. Overall, 100% of the TC clusters left the channel through the central outlet together with the TCs. In addition, viscous drag effects were observed on the WBCs in some experiments, which were partially entrained by the TCs during their acoustic drift motion toward the pressure node.

### 3.3. Cell Viability after Separation through the THINUS-Chip

**Figure 7:**
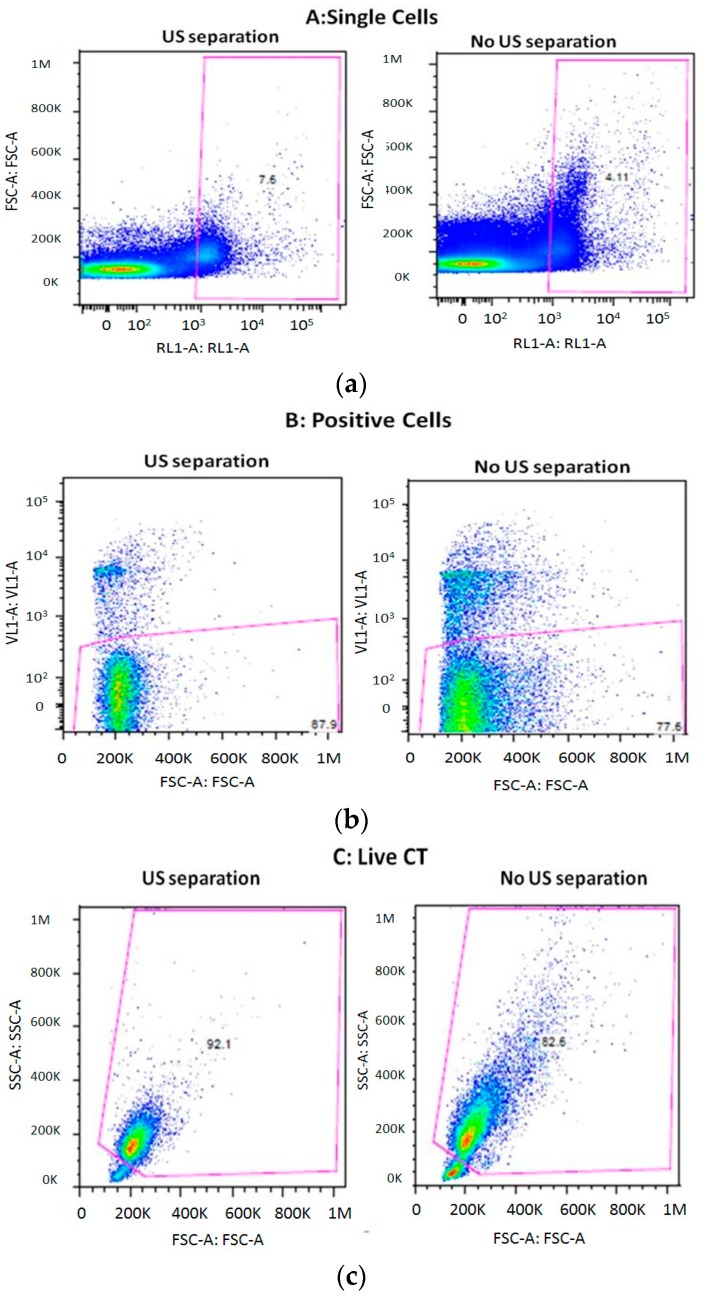
Analysis of cell viability by flow cytometry with and without US chip cell separation*.* (**a**) Single non-aggregated cells were first selected for using FSC-Area (FSC-A) versus FSC-height, and DilC18-stained TC cells were then identified by excitation with a red laser and emission capture at 440/50 nm (RL1-A). (**b**) Within the DilC18-positive TC population, DAPI-negative cells (i.e., live cells) were determined by excitation with a violet laser and emission capture at 670/14 nm (VL1-A). (**c**) Size and complexity analysis of live cells using SSC-A versus FSC-A.

Cell viability was measured after the US chip separation by flow cytometry with the Attune Nxt Acoustic 4-laser cytometer (ThermoFisher Scientific, Carlsbad, CA, USA) (Figure 7). Briefly, TCs were stained prior to mixing with mononuclear cells with the fluorescent cell tracking dye, DilC18. After CTC separation with the US chip, the CTC positive fraction was analyzed by flow cytometry to determine the cell viability. In parallel, CTC-Mononuclear cell mixes that were maintained at room temperature but had not undergone US cell separation were analyzed to determine the amount of cell death caused by the separation process.

The single cell fraction was identified and selected for analysis of cell viability (Figure 7a). Single cells that were positive for DilC18 staining were then further selected (Figure 7b). Finally, cell viability of the previously selected DilC18 positive single cell population was assessed by DAPI staining (Figure 7c). Cell viability of the TC population with and without US chip cell separation was 92.1% and 82.6%, respectively. The cell viability assays carried out after acoustophoresis showed that the extracted tumor cells were unaffected by the microchip processing. In fact, the proportion of viable cells was greater after US treatment. It must be noted that the cells were taken from their optimum environment at 37 °C and 5% CO_2_ levels and exposed to room temperature conditions for the duration of the experiment of 1–2 h. This would have a detrimental effect on cell viability, although the cells exposed to US may have suffered to a lesser extent due to the increased temperature associated with US application. However, to optimize cell viability of captured cells, isolation should be performed at the 37 °C and in a 5% CO_2_ environment with culture media instead of PBS.

A comparison of advantages and disadvantages of different types of BAW and SAW devices together with our Plate-based actuated THINUS-chip are displayed in Table 2. Although SAW micro-manipulators result the most efficient for single cell handling and isolation, they are restricted to relatively high frequencies (over 9 MHz) by the physical limitations of their IDTs. BAW separators based on the resonance of the channel require rigid materials of high cost and impose fixed locations for the particle collection defined by the frequency of work. On the contrary, our Plate-like separator presents advantages of both SAW and BAW and present added advantages, such as their manufacturing very low cost, allowing even printing processes of fabrication.

Table 2 shows a comparison of advantages and disadvantages of the BAW and SAW compared to our new Plate-like ultrasonic THINUS-chip separator.

## 4. Discussion and Conclusions

A new flexible thin Ultrasonic chip “THINUS-Chip” has been developed to perform high-throughput isolation of tumor cells from peripheral blood samples, delivering the cells viable for subsequent biomolecular analyses.

The novelty of this work lies in the strategy of the geometrical and structural chip design. The SU-8 selected as the chip material and the specific surface/volume ratio of the chip allows the establishment of plate-type vibrations, and is easier to control than typical three-dimensional modes of BAW structures. It is favored by the thickness of the chip, slightly smaller than a quarter of wavelength at frequencies close to 1 MHz. In particular, the chip vibrating at *f* = 952 kHz generates a pressure node along the center of the channel, of interest to perform the TCs isolation.

Its efficiency was quantitatively analyzed under varied flow rates. We determined an optimal flow rate of *Q* = 80 µL/min to perform the highest-throughput inertial isolation of the cancer cells.

Our lab-on-a-chip microfluidic setting offers an efficient device for the isolation of viable CTs with low-cost production and very simple geometry, with a separation efficiency on the tumor cell isolation over 70% within the range of flow rates tested, i.e., between 50 µL/min (3 mL/h) and 100 µL/min (6 mL/h). They were variable with the flow rate, reaching maximal values over 80% at *Q* = 80 µL/min (4.8 mL/h). These results demonstrated that the ability of the chip to perform TC isolation decreased dramatically at higher flow rates and poorer results were obtained over these flow conditions. In particular, TC extraction below 68% and low purity rates (64%) were obtained at a flow rate of 200 µL/min, which were discarded for the analysis.

Our acoustic device, THINUS-Chip, addresses some deficiencies of other microfluidic technologies developed for cellular separation, such as BAW or SAW: it allows the selection of the frequency of work without restrictions and presents stability of the pressure node along the center of the channel to collect the cells. In addition, this device presents a low cost of fabrication and its simple design and polymeric material opens the door to future 3D printing methods for its manufacture. The THINUS-chip presents the first disposable acoustic tool to isolate the TCs with a high potential applicability for clinical tests.

## Figures and Tables

**Table 1:** Range of variability assumed for the size and density of WBCs and TCs.

Cells	Density *ρ* (kg/m^3^)	Size (µm)
WBCs	1060 ≤$\;\rho_{l}\;$≤ 1100	7 ≤ *R_L_* ≤ 15 7–8 μm and 12–15 μm Lymphocytes,10–12 μm Neutrophils and Eosinophils, 12–15 μm Basophils
Pancreas CTs	1050 ≤$\;\rho_{CT}\;$≤ 1080	10 ≤ *R_CT_* ≤ 25

**Table 2:** Comparison of properties of BAW, SAW and PAW microfluidic devices to perform cell separation.

Chip	Frequency	Advantages	Disadvantages
BAW devices chip resonance	Not restrictions	- High efficiency of separation at low energy cost (imaging power levels) - High frequency versatility	- Low acoustic stability (pressure node-frequency)
BAW devices channel resonance	Not restrictions	- High efficiency of separation at low energy cost (imaging power levels)	- Require rigid materials (high reflectivity of channel walls) - fixed cell collection locations - strict parallelism of walls
SAW devices	>9 MHz	- High efficiency - Not geometrical restrictions - Not materials restrictions - Spatial versatility of the node	- Strong restriction of frequencies over 9 MHz
THINUS Plate-like devices	Not restrictions	- Not geometrical restrictions - Not materials restrictions - Spatial versatility of the node - Low cost and printing	- Requirement of the chip Symmetry - Stability of the pressure node

## Supplementary Materials

The following are available online at http://www.mdpi.com/2072-666X/9/3/129/s1, Figure S1: Number of TCs and WBCs counts through flow cytometry (Z2-Beckmann Coulter Counter) of six cell samples (200 μL-volume) extracted through the two outlets of the Locust-chip after their ultrasonic processing (*f* = 952 kHz, *V* = 28 V) at a flow rate of *Q* = 80 µL/min.
