# Supplementary material for: A Label Free Disposable Device for Rapid Isolation of Rare Tumor Cells from Blood by Ultrasounds
Source: Micromachines (Basel). 2018 Mar 15;9(3):129. doi: 10.3390/mi9030129 (PMC6187722; doi:10.3390/mi9030129)
Supplement: Supplementary file 1 [file micromachines-09-00129-s001.pdf]

Supplementary Materials: A Label Free Disposable Device for Rapid Isolation of Rare Tumor Cells from Blood by Ultrasounds

Itziar González, Julie Earl, Luis J. Fernández, Bruno Sainz, Jr., Alberto Pinto, Rosa Monge, Sonia Alcalá, Adela Castillejo, Jose L. Soto and Alfredo Carrato

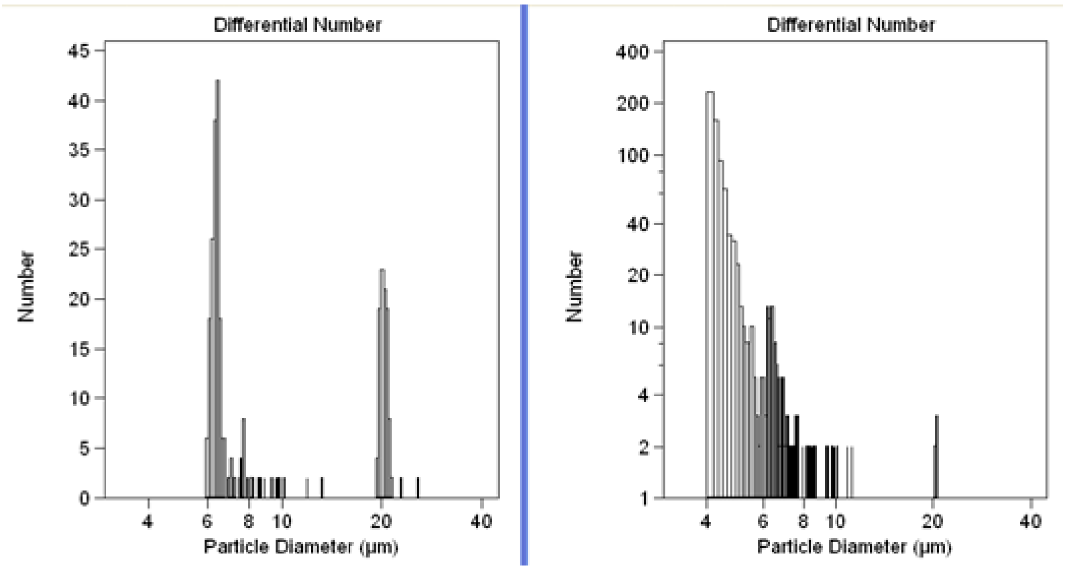

(a)

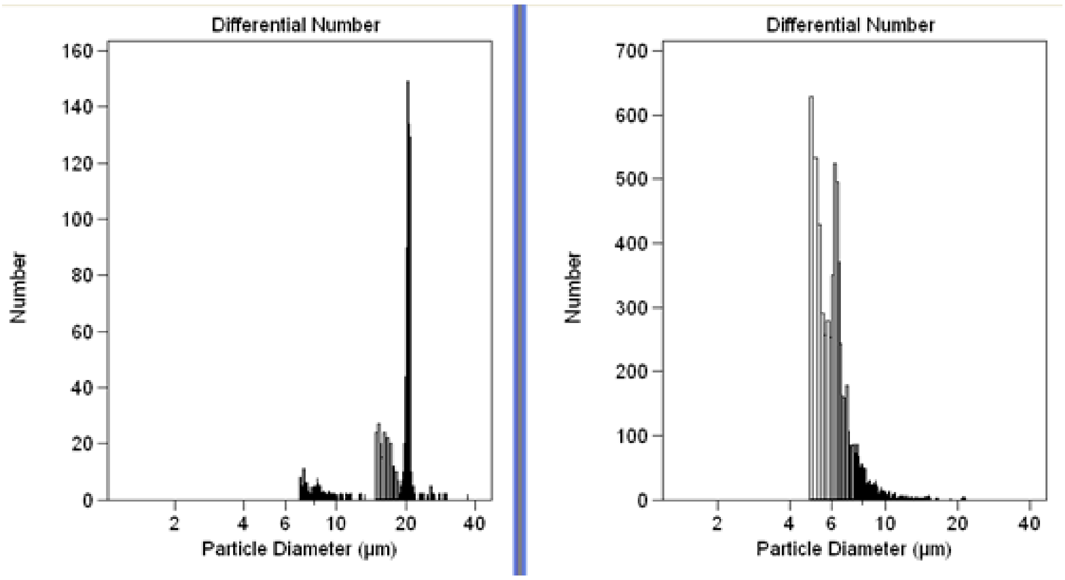

(b)

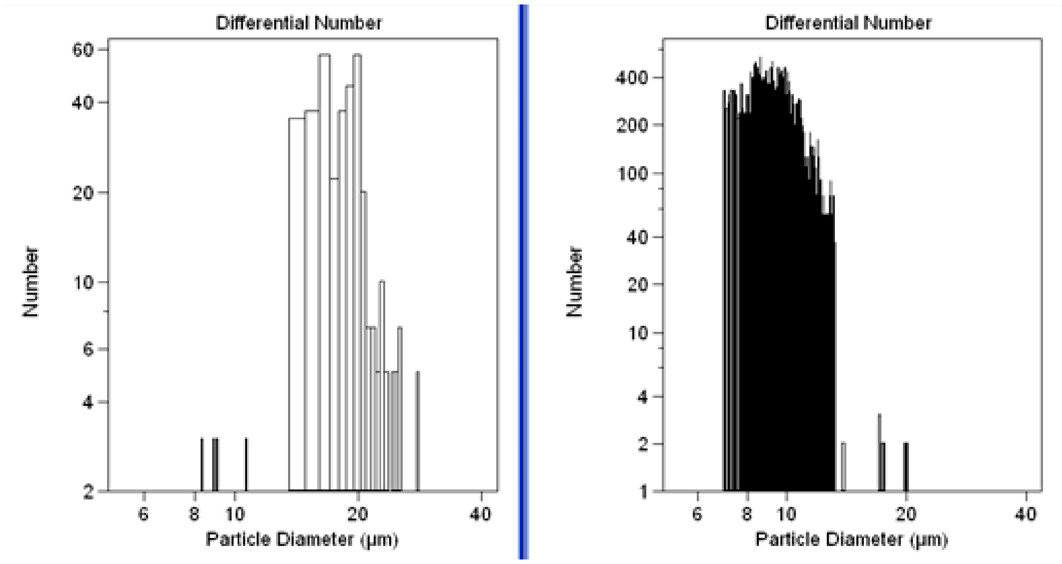
Differential Number
Number
Particle Diameter (μm)
Differential Number
Number
Particle Diameter (μm)

(c)

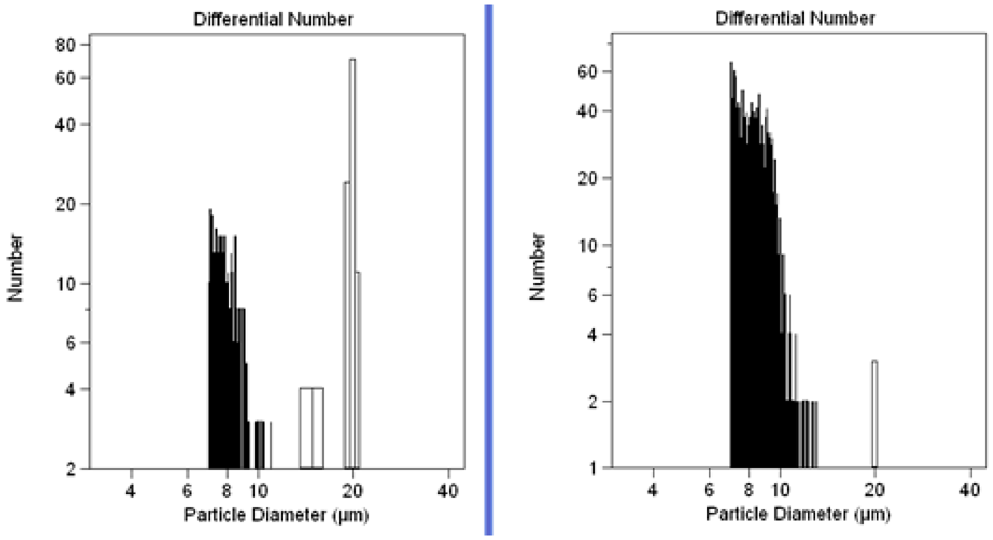
Differential Number
Number
Particle Diameter (μm)
Differential Number
Number
Particle Diameter (μm)

(d)

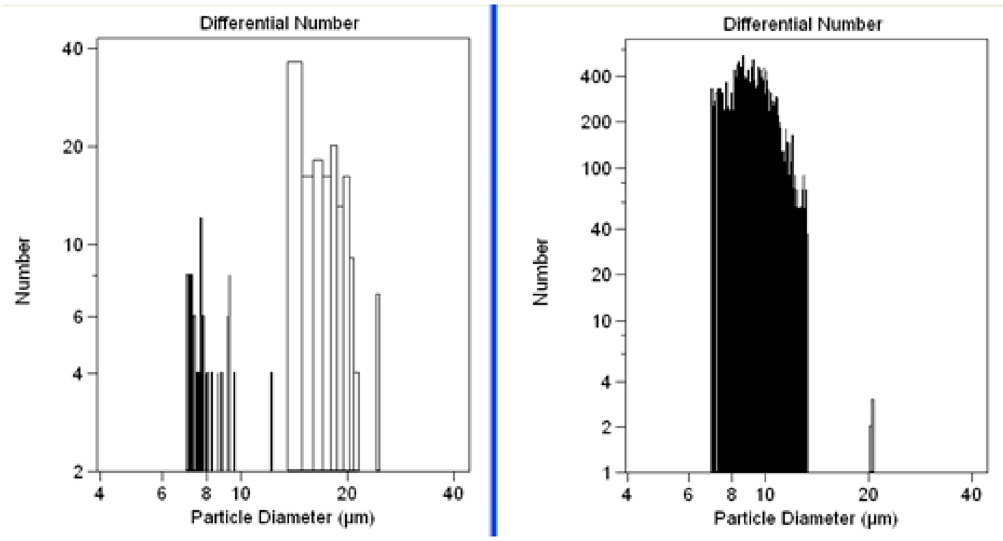
Differential Number
Number
Particle Diameter (μm)
Differential Number
Number
Particle Diameter (μm)

(e)

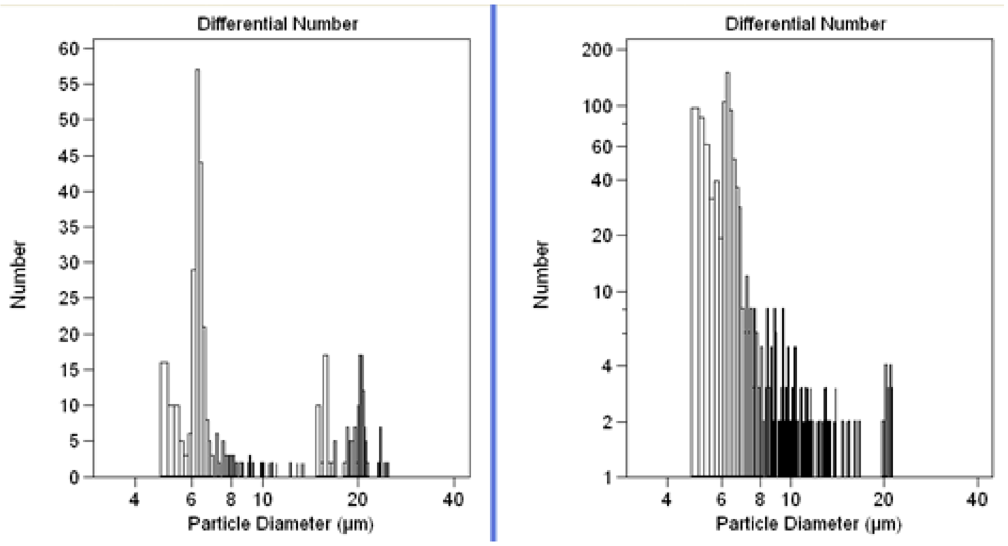

(f)

Figure S1. Number of TCs and WBCs counts through flow cytometry (Z2-Beckmann Coulter Counter) of six cell samples (200μL-volume) extracted through the two outlets of the Locust-chip after their ultrasonic processing (f = 952 kHz, V = 28 V) at a flow rate of Q = 80 μL/min.
